# Supplementary material for: DFT Study of WS2-Based Nanotubes Electronic Properties under Torsion Deformations
Source: Nanomaterials (Basel). 2023 Oct 4;13(19):2699. doi: 10.3390/nano13192699 (PMC10574366; doi:10.3390/nano13192699)
Supplement: Supplementary file 1 [file nanomaterials-13-02699-s001.zip › nanomaterials-2638747-supplementary.pdf]

Supplementary Materials

DFT Study of WS_2-Based Nanotubes Electronic Properties under Torsion Deformations

Anton V. Domnin *, Ilia E. Mikhailov and Robert A. Evarestov

Quantum Chemistry Department, St. Petersburg State University, 7/9 Universitetskaya nab., 199034 St. Petersburg, Russia; st087328@student.spbu.ru (I.E.M.); r.evarestov@spbu.ru (R.A.E.)
* Correspondence: a.domnin@sbpu.ru

Comparison of calculated properties of bulk 2H−WS2 and monolayer with experimental data

Parameters, atomic positions, and properties of the structures with rational Q nearest to the energy minima

Table S1. Properties of considered nanotubes.

(n1, n2)	ω, °	D, Å	E_{gap}, eV	E_v, eV	E_c, eV	Band gap behavior
(36, 9)	−2.857	41.342	1.84	−6.43	−4.59	Indirect
	−1.607	41.837	2.21	−6.46	−4.24	Indirect
	−1.118	41.893	2.30	−6.45	−4.16	Indirect
	−0.695	41.910	2.34	−6.45	−4.11	Indirect
	−0.326	41.916	2.37	NA	NA	NA
	0.000	41.905	2.38	−6.46	−4.08	Indirect
	0.343	41.904	2.37	−6.46	−4.09	Indirect
	0.547	41.909	2.35	NA	NA	NA
	1.353	41.914	2.23	−6.44	−4.21	Indirect
	2.143	42.015	1.98	−6.39	−4.41	Indirect
	3.025	42.129	1.62	−6.30	−4.69	Indirect
(24, 6)	−2.857	27.862	1.73	−6.40	−4.67	Indirect
	−1.607	28.206	2.02	−6.38	−4.36	Indirect
	−1.118	28.245	2.09	−6.37	−4.29	Indirect
	−0.695	28.256	2.13	−6.37	−4.24	Indirect
	−0.326	28.262	2.15	NA	NA	NA
	0.000	28.264	2.15	−6.37	−4.22	Indirect
	0.343	28.264	2.14	NA	NA	NA
	0.547	28.265	2.12	−6.37	−4.25	Indirect
	1.353	28.282	2.02	−6.36	−4.34	Indirect
	2.143	28.322	1.79	−6.32	−4.53	Indirect
	3.025	28.381	1.47	−6.24	−4.77	Indirect
(12, 3)	−2.857	14.674	1.22	−6.13	−4.91	Indirect
	−1.607	14.854	1.30	−6.04	−4.74	Indirect
	−1.118	14.877	1.32	−6.02	−4.70	Indirect
	−0.695	14.888	1.34	−6.01	−4.67	Indirect
	−0.326	14.892	1.36	−6.01	−4.65	Indirect
	0.000	14.898	1.39	−6.02	−4.63	Indirect
	0.343	14.898	1.38	−6.03	−4.65	Indirect
	0.547	14.899	1.36	−6.03	−4.67	Indirect
	1.353	14.900	1.34	−6.03	−4.69	Indirect
	2.143	14.899	1.28	−6.02	−4.74	Indirect
	3.025	14.902	1.21	−6.00	−4.79	Indirect
(8, 2)	−2.857	10.533	0.81	−5.77	−4.96	Direct
	−1.607	10.657	0.72	−5.61	−4.90	Indirect
	−1.118	10.657	0.72	−5.59	−4.87	Indirect
	−0.695	10.667	0.75	−5.59	−4.84	Indirect
	−0.326	10.674	0.79	−5.61	−4.82	Indirect
	0.000	10.679	0.83	−5.62	−4.80	Indirect
	0.343	10.683	0.86	−5.64	−4.78	Indirect
	0.547	10.685	0.88	−5.64	−4.76	Indirect
	1.353	10.693	0.82	−5.61	−4.79	Indirect
	2.143	10.699	0.73	−5.57	−4.84	Indirect
	3.025	10.699	0.69	−5.55	−4.86	Indirect

This table presents values for the diameter (D), band gap (E_{gap}), energy levels at the top of the valence band (E_v) and bottom of the conduction band (E_c), along with the behavior of the band gap.

Plots of band structure considered nanotubes under torsion deformation.

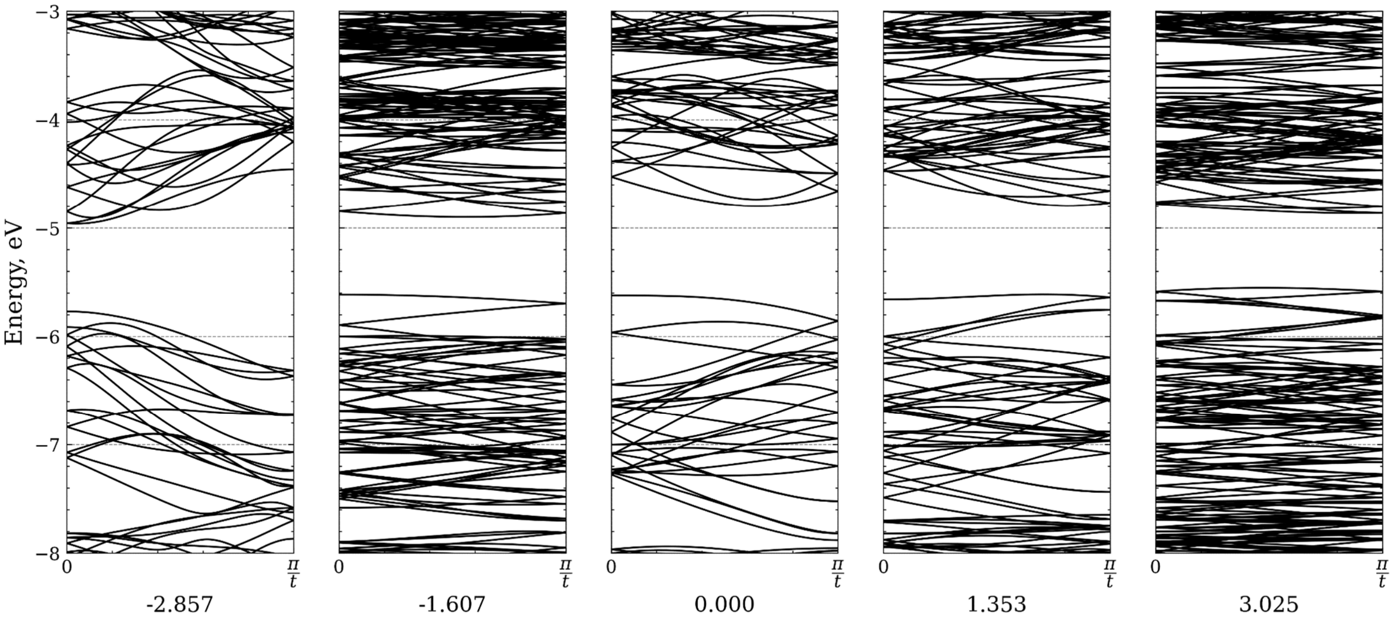

Figure S1. Band structures of (8, 2) nanotube for structures with different torsion angle.

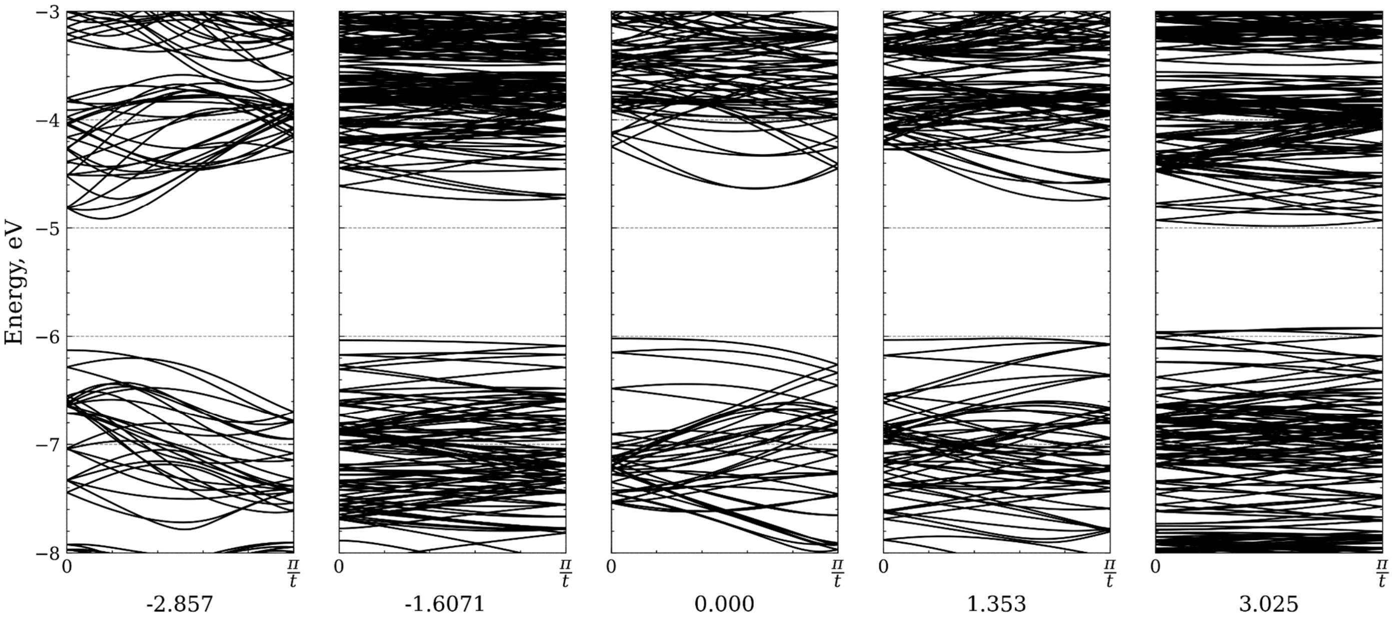

Figure S2. Band structures of (12, 3) nanotube for structures with different torsion angle.

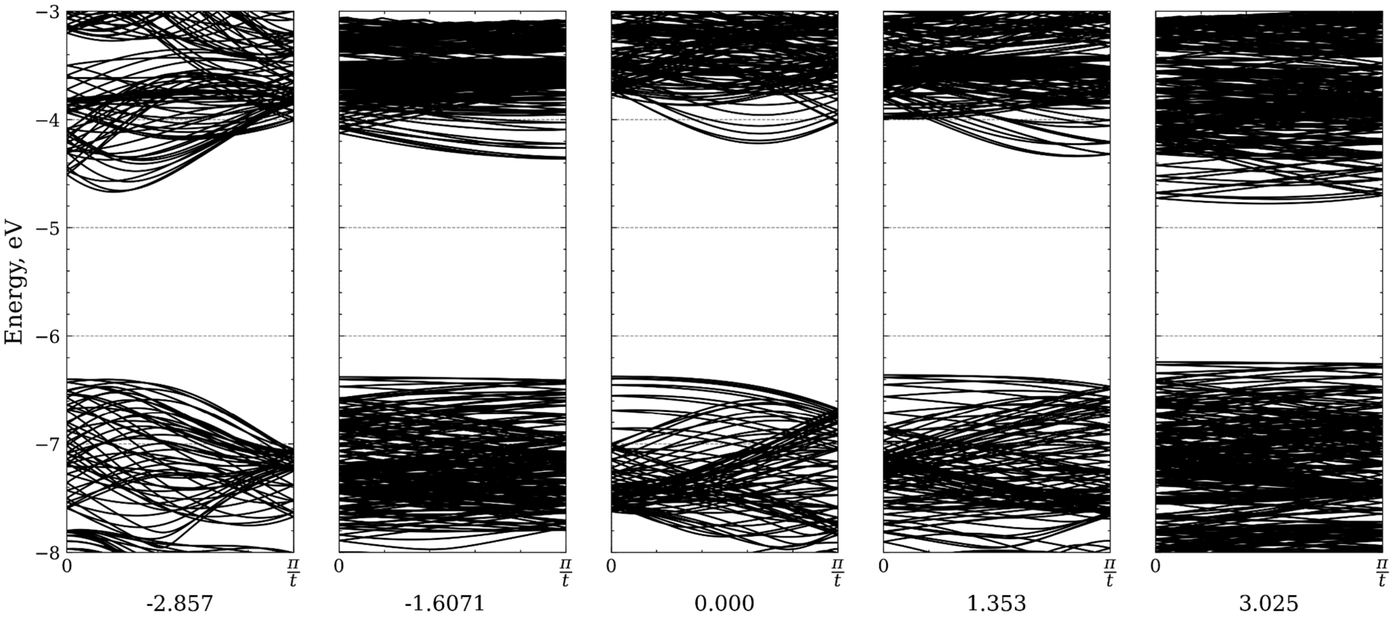

Figure S3. Band structures of (24, 6) nanotube for structures with different torsion angle.

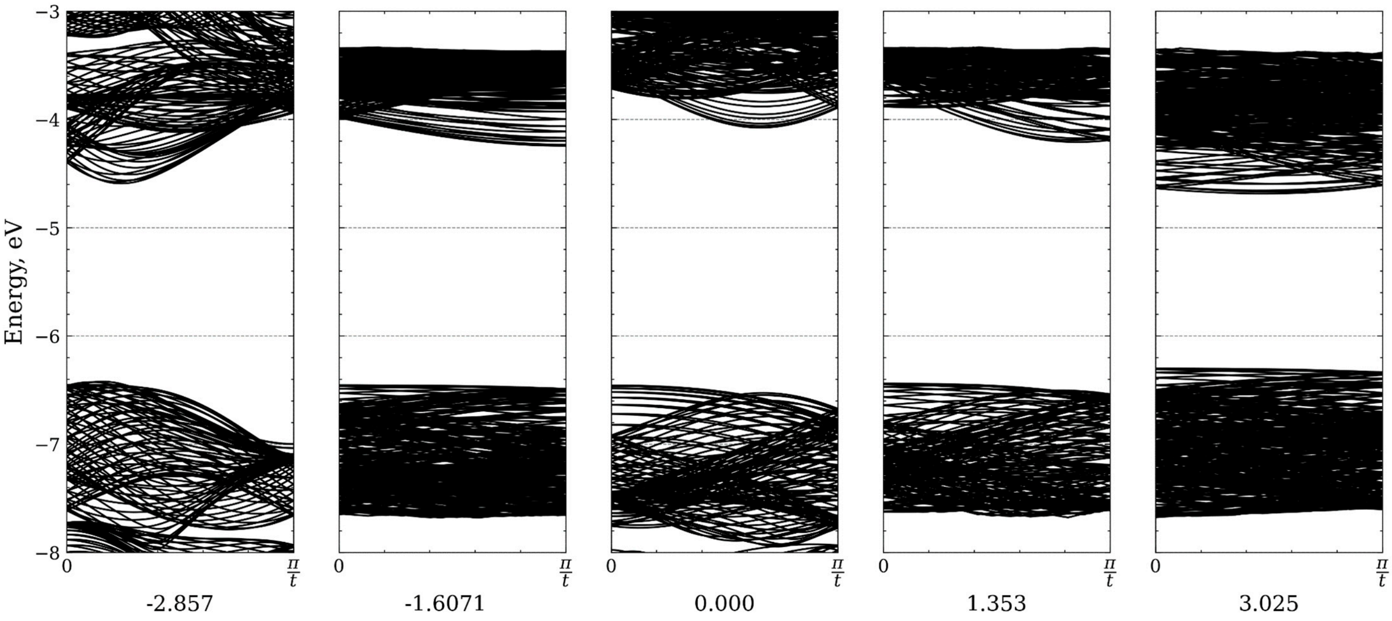

Figure S4. Band structures of (36, 9) nanotube for structures with different torsion angle.
